# The Embedding Problem for Markov Models of Nucleotide Substitution

**DOI:** 10.1371/journal.pone.0069187

**Published:** 2013-07-30

**Authors:** Klara L. Verbyla, Von Bing Yap, Anuj Pahwa, Yunli Shao, Gavin A. Huttley

**Affiliations:** 1 Computational Genomics Laboratory, John Curtin School of Medical Research, The Australian National University, Canberra, Australian Capital Territory, Australia; 2 Department of Statistics and Applied Probability, National University of Singapore, Singapore; 3 CSIRO Mathematic, Informatics and Statistics, CSIRO, Canberra, Australian Capital Territory, Australia; University of California, San Diego, United States of America

## Abstract

Continuous-time Markov processes are often used to model the complex natural phenomenon of sequence evolution. To make the process of sequence evolution tractable, simplifying assumptions are often made about the sequence properties and the underlying process. The validity of one such assumption, time-homogeneity, has never been explored. Violations of this assumption can be found by identifying non-embeddability. A process is non-embeddable if it can not be embedded in a continuous time-homogeneous Markov process. In this study, non-embeddability was demonstrated to exist when modelling sequence evolution with Markov models. Evidence of non-embeddability was found primarily at the third codon position, possibly resulting from changes in mutation rate over time. Outgroup edges and those with a deeper time depth were found to have an increased probability of the underlying process being non-embeddable. Overall, low levels of non-embeddability were detected when examining individual edges of triads across a diverse set of alignments. Subsequent phylogenetic reconstruction analyses demonstrated that non-embeddability could impact on the correct prediction of phylogenies, but at extremely low levels. Despite the existence of non-embeddability, there is minimal evidence of violations of the local time homogeneity assumption and consequently the impact is likely to be minor.

## Introduction

DNA sequences are widely used to infer evolutionary relationships among species, genes, and genomes. When modelling sequence evolution, like other complex natural phenomenon, simplifying assumptions are made for efficient computation. For sequence evolution maximum likelihood estimation for a probabilistic model is most common. This is because maximum likelihood estimation is statistically consistent (provided the underlying model is identifiable). All probabilistic models of sequence evolution generally adopt a set of simplifying assumptions relating to the sequence properties and the evolutionary process to make the models computationally tractable and statistically efficient. Markov models are commonly used and make the fundamental assumption that sites evolve independently according to a Markov process. The Markov chain is often assumed to be stationary, reversible, continuous and time-homogeneous. Stationarity assumes the process is in equilibrium resulting in equivalent ancestral and stationary base frequencies. Reversibility presumes the process appears identical when moving forward or backward in time, resulting in symmetric joint frequencies of ancestral and descendant bases. Continuity assumes the time interval between successive substitutions can be any positive number. Time-homogeneity means substitution rates at any time are fixed, described by a rate matrix (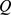). A globally homogeneous process assumes that all branches share the same rate matrix. To relax the assumption of global time-homogeneity, some approaches now allow separate substitution rate matrices for each branch of the tree (local time homogeneity).

These computationally useful assumptions are in contrast to what is understood as the biological reality; for example, compositional changes in base frequencies are a feature of sequence evolution [1]. When assumptions are violated and the model cannot account for the confounding signals in the data, the inferred results have been demonstrated to be inconsistent and erroneous (e.g. [2]–[11]). Such studies revealed violations of the assumption(s) tested i.e. model misspecification, and demonstrated that these violations increase error rates and can result in the inference of the wrong tree topology and evolutionary distances. Despite these findings, when examining other assumptions, the validity of the presumption of local time-homogeneity has yet to be explored and so is examined in this study.

Sequences may have evolved from a homogeneous or inhomogeneous, time-continuous or discrete process. However because modelling a time-continuous inhomogeneous process is statistically infeasible, homogeneity is assumed when the widely implemented time-continuous models are used. The alternative can, to some extent, be captured by a discrete process. This alternative process could be time-continuous but inhomogeneous or simply discrete. The most commonly implemented discrete model was proposed by [12]. This model is referred to in this study as the BH model. Their approach makes only the assumption of process-homogeneity, but does not assume continuity, time-homogeneity (local or global), reversibility or stationarity. The BH model formulation has no instantaneous rate matrices, 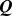, but uses an independent transition probability matrix, 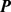, for each edge. If the process captured in the transition matrix (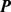) is a discrete manifestation of an underlying time homogeneous and continuous Markov chain, then relationship
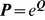
(1)holds (where 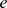 is the matrix exponential). If the relationship holds true then the process is said to be embeddable and can in fact be modelled as continuous and time homogeneous. Conversely if the assumption of homogeneity is violated, the relationship in (1) does not hold and the underlying process is said to be non-embeddable. It could be non-embeddable because the process is discrete or continuous and time-inhomogeneous such that a 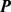 exists where, for example, 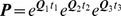 describes the process but there is no valid instantaneous rate matrix satisfying (1).


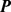
 and 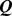 matrices satisfying (1) must have certain characteristics in order to be valid Markov matrices. The substitution rate matrix 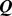 is normally constrained to satisfy 3 conditions. It must have non-negative off-diagonals 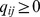 for 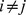 where 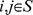 and 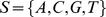, the rows must sum to 0, 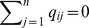 for 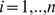 (where 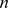 is the dimension of 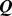, 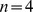 for nucleotides) and 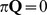 where 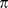 are the base frequencies. The transition probability matrix 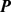 is defined to have rows that sum to 1, 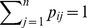 for 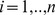. A validly defined 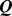 matrix will produce a valid 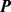 [13]. However, the reverse is not true. 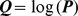, the converse of (1), can result in a valid 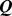, an invalid 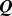 (a 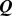 with negative off-diagonals) or be unable to produce a 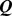 (the matrix logarithm of 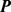 can not be calculated). In the cases where no valid 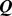 can be produced, 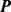 is non-embeddable and cannot be embedded into a continuous and time-homogeneous chain.

The question of how to formally determine if a transition matrix (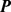) is embeddable is known as the embedding (or imbedding) problem and was first described in [14]. This study established the sufficient conditions for embeddability for a 2×2 matrix. Further investigations have been carried out into the sufficient conditions for embeddability for the 3×3 case for both time-homogeneous and non-homogeneous processes [15]–[21]. The complicating issue is that for a 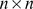 matrix where 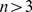 there are no simple sufficient general conditions for establishing if 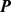 is embeddable. A set of simple steps for the 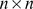 case was put forward by [22] to enable the identification of matrices that were non-embeddable. The results of this study have been widely implemented in the sociology field for analyses with Markov processes and are adopted here.

**Figure 1:**
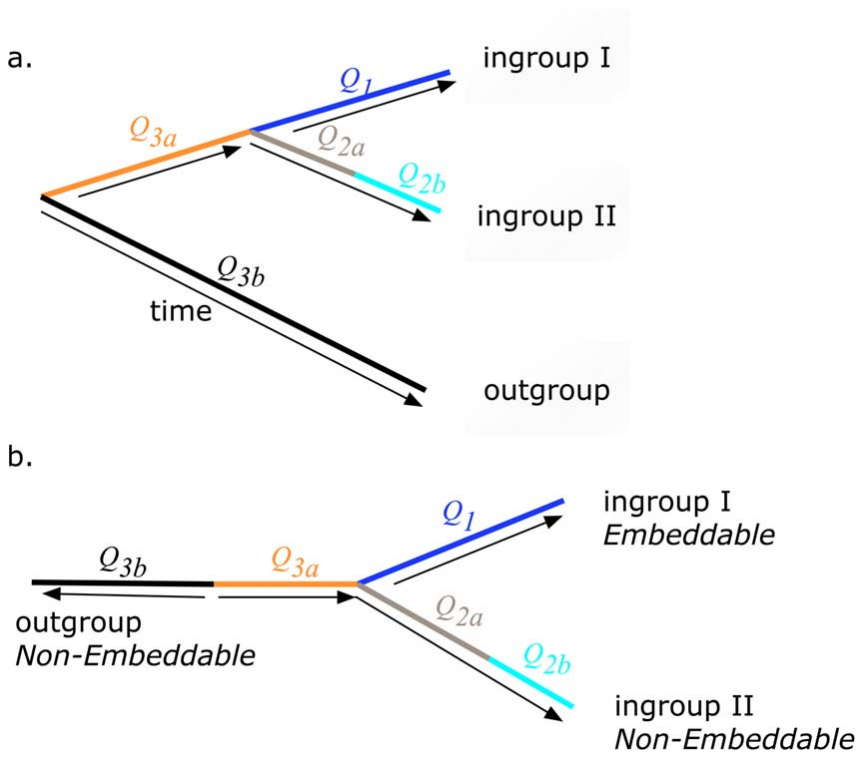
Rooted (a) and unrooted (b) phylogenetic tree with embeddable (single 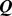) and non-embeddable (multiple 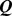) edges.

Non-embeddability will occur where there is a need for different instantaneous rate matrices 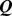 per branch (see Figure 1) caused either by a discrete process or by a time-continuous but inhomogeneous process. Evident from Figure 1 is that a natural control exists when modelling sequence evolution with an unrooted tree. On the outgroup edge in any unrooted tree are dual 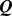 matrices reflecting that this branch contains both forward and backward time. Consequently, it is expected that if non-embeddability exists then it is likely to be found on the outgroup edge. In addition, it was suspected that non-embeddability was more likely to exist on edges with larger time depths.

The aim of this study was first to determine if there was evidence of violations of the assumption of local time-continuous homogeneity through establishing the existence of non-embeddability. Secondly, the study sought to determine the extent and effect of the occurrence of non-embeddability when modelling evolutionary processes with a time-homogeneous continuous Markovian model. Species triads from across the tree of life were analysed for evidence of non-embeddability. Due to unequal selection and mutagenesis pressures at the different codon positions, protein coding alignments were divided into codon positions. At each codon position, all edges in each sequence triad were tested separately for evidence of non-embeddability by allowing each individual edge to have independent 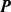 and 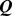 matrices. The evidence of non-embeddability was gathered by assessing the characteristics of the 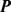 (and 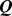) matrices. A parametric bootstrap approach comparing the log-likelihood ratio statistic (logLR) was then used to determine if there was a difference in model fit for those alignments where a non-embeddable 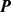 was identified. A significant difference in model fit confirmed the violation of the assumption of time-homogeneity and that the process was non-embeddable. Once the study had demonstrated the existence of non-embeddability, the effect of non-embeddability, and consequently the violation of the assumption of local time-homogeneity, on phylogenetic reconstruction was explored.

## Materials and Methods

### Data

Four diverse datasets were used to test for the existence of non-embeddability across the tree of life. The characteristics of the datasets are summarised in Table 1. All datasets contained orthologs for at least three taxa and had distinct outgroup(s). Species triads were employed due to the consistency property of maximum likelihood tree reconstruction which showed that the joint distributions of three terminal nodes are enough to determine the full model [23]. For each data set, the sequences were aligned with all ambiguous sites and gaps removed using the progressive aligner from PyCogent [24]. Protein coding sequences were separated into codon positions due to the different selection pressure at each location [25], [26]. The identification of non-embeddability at a particular codon position will give an indication of whether the violation of the continuous time-homogeneous assumption is caused by a mutation or selection rate change. The datasets span both the vertebrates and microbes in order to fully investigate the existence of non-embeddability.

**Table 1:** Summary of Datasets.

Data Set	Taxa*^a^*	Number of Alignments	Sequence Length (bp)	Total Tree length *^b^*
D1: Nuclear protein- coding genes	O,M,H	8193	>300	1.7081
	O,M,R	8014	>300	1.5622
	H,M,R	8394	>300	0.6890
D2: Mitochondrial protein-coding genes	M, H, O	11	67–598	3.7267
D3: Primate introns	C, H, Ma	62	>50,000	0.0763
D4: Microbial protein- coding genes	bad, bas, bba, bbu, bpn, bvu, cjk, dps, eca, ent, kra, lla, lre, mgi, mle, mta, ppe, pth, sma, wsu *^d^*	1	591–867	1.935

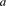
 – C: Chimpanzee, H:Human, M:Mouse, Ma:Macaque, O:Opossum, R:Rat, bad:Bifidobacterium adolescentis, bas: Buchnera aphidicola Sg, bba:Bdellovibrio bacteriovorus, bbu:Borrelia burgdorferi B31, bpn: Candidatus Blochmannia pennsylvanicus, bvu: Bacteroides vulgatus, cjk:Corynebacterium jeikeium, dps:Desulfotalea psychrophila, eca:Pectobacterium atrosepticum, ent:Enterobacter sp. 638, kra:Kineococcus radiotolerans, lla:Lactococcus lactis subsp. lactis IL1403, lre:Lactobacillus reuteri DSM 20016, mgi:Mycobacterium gilvum, mle:Mycobacterium leprae TN, mta:Moorella thermoacetica, ppe:Pediococcus pentosaceus, pth:Pelotomaculum thermopropionicum, sma:Streptomyces avermitilis, wsu:Wolinella succinogenes, 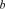 – average length from consensus tree , 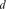 -All possible triads (1140).

### Vertebrates

The vertebrate alignments were obtained from Ensembl release 58 except for the intron dataset which was obtained from Ensembl release 50. The sampling process for this intron dataset is described in detail in [27]. The first data set (D1) was used to investigate whether elapsed evolutionary time (time depth) influenced the existence of non-embeddability. This was investigated by using three triads with varied time depth between taxa. The triads of human, mouse and opossum, had the longest time depth between all taxa with opossum functioning as the outgroup. The triad of mouse, rat and opossum had a shorter time depth between the two ingroup taxa. The final triad consisting only of Eutherian taxa (mouse, rat, human) contains the shortest time depth between all taxa with the human group functioning as the outgroup. Whether non-embeddability occurred in both the nuclear and mitochondrial genomes was explored using datasets D1(nuclear) and D2 (mitochondrial). The intronic dataset (D3) was included to examine whether sequence function (coding/non-coding) impacted upon the presence of non-embeddability. The dinucleotide model was used to analyse this dataset as it has been demonstrated to give an improved model fit for this data [27].

### Microbes

A single microbial protein-coding gene was selected to assess the extent of non-embeddability across a range of species. Twenty microbial species with differing estimated evolutionary divergence were randomly chosen from an aligned set of 197 microbial species for the gene, *translation initiation factor, IF-2*, originally extracted from the KEGG database [28]. The 197 species were originally selected as they had at least 500 orthologs from a set of 2226 orthologs that spanned at least 60 species. All 1140 possible triads for the 20 species for this gene were investigated for evidence of non-embeddability.

### Substitution Models

For each triad, every edge was modelled separately assuming a discrete or continuous time homogeneous process. The assumptions for each process can be found in Table 2. Two differing Markov substitution models were used to test each edge for non-embeddability. The first model (herein referred to as the mixed model) assumed a continuous and time homogeneous process on the edge being tested for non-embeddability, while all other edges in this model and all edges in the second model (the discrete model) were modelled as discrete using the BH model (see Table 3). The models for the discrete and continuous processes applied to individual edges are described in the next section. All edges in both models were assumed to have a process that was independently and identically distributed, 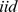. If the process on an edge being tested was non-embeddable (i.e. generated by multiple 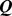 see Figure 1) then a discrete model not assuming time-homogeneity for that edge will produce an non-embeddable 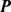 and have a better model fit than a mixed model. Conversely if a single 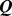 accurately describes the underlying process on an edge then the discrete model will generate a embeddable 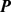 and will have the same model fit as the mixed model.

**Table 2:** Markov Process Assumptions for an Edge.

Assumption	Continuous	Discrete (BH)
Time- Homogeneity	√	X
Reversibility	X	X
Stationary	X	X
Independent Sites	√	√

**Table 3:** Summary of The Two Markov Models.

Edge	Tested*^a^*	Mixed Model	Discrete Model
1	Yes	Continuous *^b^*	Discrete
2	No	Discrete	Discrete
3	No	Discrete	Discrete

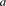
 – tested for non-embeddability, 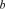 – Assumption of local time-homogeneity.

**Figure 2:**
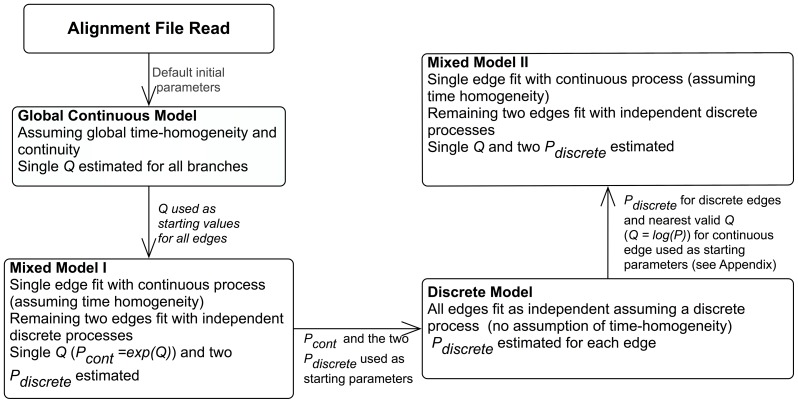
Testing scheme.

Maximum likelihood was used to obtain the model fit and parameter estimates for both models. The likelihood function was optimised using two optimisation approaches available in the PyCogent toolkit; the Powell method [29] and simulated annealing (global optimisation) [30]. Initial parameter estimates for the mixed model were provided by a continuous, globally time homogeneous model to help ensure optimisation. The parameter values for all edges from the mixed model were subsequently used as initial starting values for the discrete model. Providing initial parameter estimates to BH models is suggested as the algorithm is known to converge to local maxima if the initial values used for the 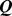 matrices are not diagonally dominant or if the rate of convergence is too slow [31]–[33]. To check the stability of the original mixed model parameter estimates, parameter estimates from the discrete model were used as the starting values for a second optimisation of the mixed model. If a non-embeddable 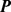 matrix was found using the discrete model, then there was no valid 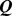 matrix to use as starting parameters for the mixed model. Consequently, the nearest valid 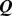 for this edge was found by minimising the Frobenius norm of the difference between the non-embeddable 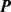 and estimated nearest embeddable 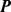 i.e. a 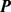 that produces a valid 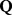 (see Appendix A in Supporting Information S1). The logLR for the second optimisation of the mixed model was then compared with the original estimate to ensure stability and correct optimisation. The overall testing scheme is displayed in Figure 2.

### Continuous and Discrete Markov Processes

There were 39 unknown parameters in both the mixed and fully discrete nucleotide models (12 for each 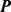 and 3 parameters for the base frequencies i.e. 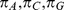 where 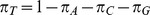) and 735 unknown parameters for the dinucleotide case (240 for each 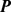 and 15 parameters for the dinucleotide frequencies). Each 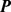 was produced either assuming a discrete (BH model) or continuous process. Under the BH model, a 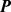 matrix is calculated based on the joint probability distribution of the nucleotides at each end of an edge. The likelihood was maximised using a system of iterative equations for the joint probability distributions along each edge. This approach for an unrooted triple of sequences from three species is well described in [33] and in more general terms in [32].

For a continuous process, the time-homogeneous transition probabilities, 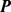, are governed by the forward Kolmogorov equation and the initial condition:
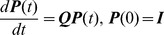
(2)where 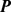 and 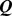 are 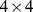 matrices and 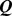 has the structure



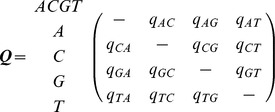
where



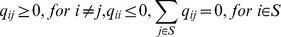



The functions, 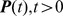, which are solutions of (1), comprise the transition matrices of a time homogeneous continuous Markov chain. Solutions for (2) are given by:
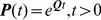
(3)


If the time factor is removed then (3) becomes 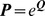. Constrained optimisation is used to find a valid 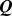 [34]. It is then exponentiated to find an estimate of 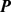.

### Embeddability

Let 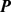 be a time-homogeneous transition matrix for a discrete Markov chain with finite states. If 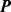 is a discrete manifestation of a continuous and time-homogeneous Markov chain, then 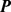 is said to be embeddable and consequently 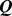 is said to generate 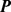 such that, as in (1):
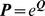



However, this only holds true if 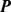 can be embedded in a continuous Markov process. Whether 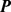 is embeddable can be determined by mathematically assessing the characteristics of the 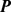 and 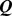 matrices.

The steps for determining if the transition matrix, 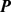, is embeddable where 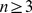 and adopted in this study are as follows:

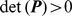
 [22], [34], [35].
2. The negative eigenvalues of 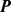 must have even algebraic multiplicity [14], [16], [22].
3. Any complex eigenvalues of 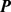 must occur in conjugate pairs [16], [22].
4. All eigenvalues of 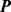 must lie inside a ‘heart shaped’ region in the complex plane whose boundary is the curve 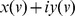 where
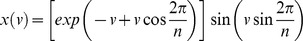


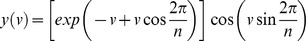
with 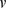 restricted to 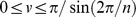 [22], [36].
5. Examine 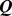 for negative off-diagonals [37].

These steps are necessary but not sufficient and were the first stage of identifying non-embeddability in this study. This stage produced a reduced set of alignments for which an edge had a non-embeddable 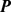 identified. This set of alignments was then examined using a parametric bootstrap.

### Parametric Bootstrap

A parametric bootstrap scheme was implemented for two reasons. The first was because the distribution of the logLR is unknown. This is due to the identical number of parameters in each model (mixed and discrete). Therefore to determine whether there is a significant difference in model fit, a parametric bootstrap is required to establish the null distribution for the logLR. Secondly, the parametric bootstrap will also enable the determination of whether a non-embeddable 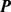 matrix found when examining the characteristics of 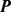 and 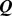 is caused by a truly non-embeddable process. Maximum likelihood estimates of 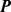 may identify non-embeddability just because substitutions are stochastic and computational precision issues could cause an non-embeddable 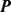 despite the underlying process being time homogeneous.

The parametric bootstrap was used to test the null hypothesis, 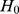: The process can be embedded in a continuous chain and there is no violation of the time-homogeneous assumption i.e. the mixed model produces the same model fit, versus the alternative hypothesis, 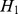: The process is not embeddable in a continuous chain and there is a violation of the time-homogeneous assumption i.e. the discrete model has a better model fit than the mixed model. 1000 parametric bootstrap samples were simulated under the null hypothesis (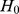) to establish the distribution of the test statistic for each alignment. This was carried out only for edges of alignments where a non-embeddable matrix was identified. The bootstrap testing scheme is outlined as follows:

1. Determine the logLR (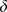) for the observed alignment 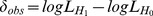, where 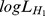 is the log likelihood produced by the discrete model and 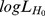 is the log likelihood indicated by the mixed model (with a continuous process fitted for the edge that produced a non-embeddable matrix).
2. Generate 1000 bootstrapped datasets of the alignment under the null hypothesis (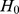).
3. Calculate the difference in logLR statistics 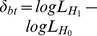 for each bootstrap data set.
4. Calculate the proportion 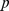 of times that 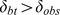.
5. Reject the null hypothesis, 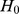, when the proportion 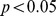 and confirm non-embeddability for the edge of the alignment tested.

In addition to the negative control described above, a positive control was also implemented. The parameter estimates from an randomly selected alignment found to have a non-embeddable edge was used to generate 1000 bootstrapped samples (i.e. under the alternative hypothesis). Each sample was 1000 base pairs in length. These were then tested for evidence of non-embeddability by examining the matrix characteristics and using the parametric bootstrap. The number of simulated alignments identified as non-embeddable was then calculated to determine the power of the procedure to correctly classify alignments generated by a non-embeddability process.

### Phylogenetic Reconstruction

One important aim when modelling sequence evolution is to establish the correct relationship between the sequences and construct an accurate phylogenetic tree. Despite finding evidence that a model fits the data better than an alternative model, this does not always translate into different results when constructing the most probable trees [31]. To assess whether incorrectly modelling a process as time-homogeneous (and therefore embeddable) has an effect on phylogenetic reconstruction, a fully general continuous model assuming local time-homogeneity for all edges and the discrete BH model were used to find the most probable tree using maximum likelihood. The two models were used as implemented in the PyCogent toolkit with the continuous model (“General” model in PyCogent) having 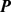 matrices for all edges set as independent to allow the assumption of local time-homogeneity (default setting is for global time-homogeneity). Datasets D1 and D3 were used. In the mammalian dataset (D1), 8005 alignments with sequences for the tetrad of mouse, rat, human and opossum were separated into codon positions. The second dataset contained 4845 tetrads formed using dataset D3 for the 20 species and gene *IF2*. Each alignment at all codon positions had a minimum length of 300 bp and the number of variable sites was required to be at least ten percent of total number of sites. This was to limit the possibility of incorrectly finding differences between models caused by a lack of information. For each tetrad the most probable tree was predicted using each model. Finding a difference in the predicted most probable trees will indicate a violation of the assumption of local homogeneity (and therefore non-embeddability of the process) can cause biases in phylogenetic construction.

The most probable tree was first estimated using the implemented ML trex method [38] in PyCogent. In cases where the models predicted a different tree for the same tetrad, the optimisation of the models was checked by fitting the complete models for two most probable trees in PyCogent. The total number of inconsistencies between the predicted phylogenies were then calculated for the separate codon positions.

## Results

Evidence of non-embeddability was found in all 4 datasets analysed. The number of non-embeddable matrices and number of non-embeddable processes (where the null hypothesis was rejected in the parametric bootstrap) for each alignment and triad examined are shown in Tables 4–9. The assessment of the number and magnitude of negative off-diagonal elements when testing for non-embeddable matrices revealed an extremely high number of very small negative elements. This was most likely due to precision and sampling and thus a threshold of −0.1 for off-diagonal elements was used to declare non-embeddability for a 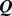 matrix. This was an arbitrary threshold based on inspection of the results.

**Table 4:** Non-Embeddability – D1 Human, Rat, Mouse Triad (8394 Alignments).

		STEPS ^a^						
Edge	Codon position	1	2	3	4	5	NE*^b^* Matrices	NE Processes*^c^*
Human	1	0	0	0	0	0	0	0
	2	0	0	0	0	0	0	0*^d^*
	3	16	16	0	3	91	107	6 (5.6)
Mouse	1	0	0	0	0	0	0	0
	2	0	0	0	0	0	0	0
	3	1	1	0	0	0	1	0
Rat	1	0	0	0	0	0	0	0
	2	0	0	0	0	0	0	0
	3	0	0	0	0	2	2	0

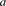
 Steps to identify Non-embeddability 1. 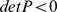, 2. Negative eigenvalues have odd algebraic multiplicity, 3. Complex eigenvalues occur in non-conjugate pairs, 4. The set of eigenvalues, 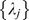, lie outside the region in the complex plane, 5. 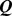 – negative off-diagonals – threshold −0.1, 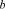 NE =  Non-Embeddable, 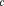 No. rejections of 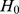 from parametric bootstrap scheme with a p-value 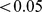 (percentage of total tests), 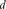 1 Alignment failed to find stable estimates.

**Table 5:** Non-Embeddability – D1 Opossum, Rat, Mouse Triad (8014 Alignments).

		STEPS *^a^*						
Edge	Codon position	1	2	3	4	5	NE*^b^* Matrices	NE Processes *^c^*
Opossum	1	0	0	0	0	4	4	0
	2	0	0	0	0	0	0	0*^d^*
	3	117	119	0	26	638	777	43 (5.5)
Mouse	1	0	0	0	0	0	0	0
	2	0	0	0	0	0	0	0
	3	1	1	0	1	2	2	0
Rat	1	0	0	0	0	0	0	0
	2	0	0	0	0	0	0	0
	3	0	0	0	0	1	1	0

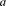
 Steps to identify Non-embeddability 1. 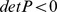, 2. Negative eigenvalues have odd algebraic multiplicity, 3. Complex eigenvalues occur in non-conjugate pairs, 4. The set of eigenvalues, 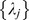, lie outside the region in the complex plane, 5. 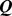 – negative off-diagonals – threshold −0.1, 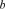 NE =  Non-Embeddable, 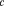 No. rejections of 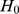 from parametric bootstrap scheme with a p-value 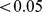 (percentage of total tests), 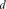 1 Alignment failed to find stable estimates.

**Table 6:** Non-Embeddability – D1 Opossum, Mouse, Human Triad (8194 Alignments).

		STEPS *^a^*							
Edge	Codon position	1	2	3	4	5	NE*^b^* Matrices	NE Processes *^c^*	
Opossum	1	3	3	0	3	4	7	1*^d^*	
	2	0	0	0	0	0	0	0	
	3	73	76	0	40	478	547	40 (7.3)	
Human	1	1	1	0	1	0	1	0	
	2	0	0	0	0	0	0	0	
	3	24	24	0	14	75	99	12 (12.1)	
Mouse	1	0	0	0	0	0	0	0	
	2	0	0	0	0	0	0	0	
	3	20	20	0	19	87	108	5 (4.6)	

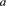
 Steps to identify Non-embeddability 1. 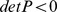, 2. Negative eigenvalues have odd algebraic multiplicity, 3. Complex eigenvalues occur in non-conjugate pairs, 4. The set of eigenvalues, 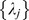, lie outside the region in the complex plane, 5. 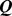 – negative off-diagonals – threshold −0.1, 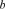 NE =  Non-Embeddable, 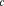 No. rejections of 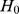 from parametric bootstrap scheme with a p-value 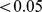 (percentage of total tests), 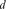 1 Alignment failed to find stable estimates.

**Table 7:** Non-Embeddability-D2 (Opossum) Mitochondrial Protein coding genes (11 Alignments).

		STEPS *^a^*						
Edge	Codon position	1	2	3	4	5	NE*^b^* Matrices	NE Processes *^c^*
Opossum	1	0	0	0	0	1	1	0
	2	0	0	0	0	0	0	0
	3	1	1	0	1	8	9	0*^d^*
Mouse	1	0	0	0	0	1	1	0
	2	0	0	0	0	0	0	0
	3	2	2	0	0	5	7	1*^d^*
Human	1	0	0	0	0	1	1	0
	2	0	0	0	0	0	0	0
	3	1	1	0	0	8	9	0*^d^*

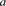
 Steps to identify Non-embeddability 1. 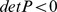, 2. Negative eigenvalues have odd algebraic multiplicity, 3. Complex eigenvalues occur in non-conjugate pairs, 4. The set of eigenvalues, 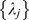, lie outside the region in the complex plane, 5. 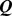 – negative off-diagonals – threshold −0.1, 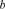 NE =  Non-Embeddable, 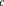 No. rejections of 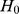 from parametric bootstrap scheme with a p-value 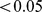 (percentage of total tests), 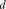 3 Alignments failed to find stable estimates.

**Table 8:** Non-Embeddability – D3: Primate Introns Dinucleotide Model (62 alignments).

	STEPS *^a^*						
Edge	1	2	3	4	5	NE*^b^* Matrices	NE Processes *^c^*
Macaque	0	0	0	0	16	16	5 (31.3)
Human	0	0	0	0	0	0	0
Chimpanzee	0	0	0	0	0	0	0

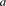
 Steps to identify Non-embeddability 1. 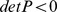, 2. Negative eigenvalues have odd algebraic multiplicity, 3. Complex eigenvalues occur in non-conjugate pairs, 4. The set of eigenvalues, 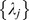, lie outside the region in the complex plane, 5. 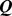 – negative off-diagonals – threshold −0.1, 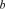 NE =  Non-Embeddable, 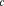 No. rejections of 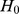 from parametric bootstrap scheme with a p-value 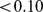 (percentage of total tests).

**Table 9:** Non-Embeddability- D4 Microbial Protein Coding Gene (1140 Triads).

	STEPS ^a^						
Codon position	1	2	3	4	5	NE*^b^* Matrices	NE Processes*^c^*
1	0	0	0	0	2	2	0
2	0	0	0	0	0	0	0
3	574	591	0	470	1052	1122	27*^d^*

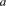
 Steps to identify Non-embeddability 1. 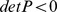, 2. Negative eigenvalues have odd algebraic multiplicity, 3. Complex eigenvalues occur in non-conjugate pairs, 4. The set of eigenvalues, 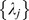, lie outside the region in the complex plane, 5. 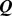 – negative off-diagonals – threshold −0.1, 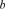 NE =  Non-Embeddable, 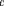 No. rejections of 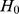 from parametric bootstrap scheme with a p-value 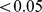, 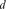 584 Alignments failed to find stable estimates.

### Vertebrates

The results for the three Mammalian triads from the nuclear protein-coding dataset (D1) are presented in Tables 4–6. The number of non-embeddable processes varied across the three triads although it almost always occurred at the third codon position. For the Eutherian triad, only a small number of non-embeddable matrices and processes were identified on the human (outgroup) edge at the third codon position. For the mouse, rat, opossum triad again non-embeddability was identified only on the outgroup edge (opossum) at the third position. The final triad of mouse, human and opossum had non-embeddable processes identified on all edges at the third codon position. A single non-embeddable process was identified at the first position on the outgroup (opossum) edge. In the mammalian mitochondrial protein coding data (D2), for a single alignment the mouse edge showed evidence of non-embeddability (Table 7).

As found in the vertebrate protein coding datasets, non-embeddability was identified in the primate intron dataset (D3). Non-embeddability was indicated on the Macaque (outgroup) edge by inspecting the matrices for 16 of the 62 alignments analysed (see Table 8). Due to the time constraints and computational demands caused by use of the dinucleotide model, only 100 parametric bootstrap replicates were run. Consequently, significant evidence for non-embeddability was declared in parametric bootstrap when there were fewer than ten parametric bootstrap replicates with a logLR greater than the original logLR (p-value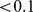) as 1000 bootstraps are considered the minimum required to declare significance with a p-value of 0.05 [39]. The use of the slightly less stringent threshold (p-value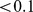) allows for the fact that 100 samples are not fully representative of the true null distribution. Of the 16 alignments with non-embeddable matrices, 5 were also found to have nominally significant evidence from the parametric bootstrap for non-embeddability using a 0.1 p-value. However should a p-value of 0.05 be used, two alignments would still indicate non-embeddability with this limited number of samples. No evidence of non-embeddability was indicated on either of the other two edges in any alignments.

### Microbes

The Microbial data provided the highest number of non-embeddable matrices with 716 triads having all three edges showing evidence of non-embeddability (Table 9). A further 311 triads had two edges showing evidence of non-embeddability and 95 triads had a single edge where a non-embeddable matrix was identified. However, only a total of 27 edges were found to have non-embeddable processes in the parametric bootstrap. Note that there were significant convergence issues during the parametric bootstrap with the mixed model for 584 triads failing to find stable estimates. While the mixed model appeared to converged to a set of parameter estimates within the set number of iterations (100 K), repeating the model fit revealed different parameter estimates. This indicated that the model may have been converging to different local maxima. In contrast, despite considering different starting parameters, the discrete model was able to converge to the same parameter estimates. This failure of the mixed model to converge meant that the parametric bootstrap was unable to determine if assuming discrete process resulted in an improved model fit over the mixed model.

As this data set contained triads constructed for a single gene across multiple microbial species, to ensure that the results was not an artifact of this particular alignment a second gene, *nusA* (*N utilization substance protein A*), was similarly analysed. The results for the non-embeddability analysis revealed almost identical results (779, 318 and 95 triads had non-embeddable matrices established on 3, 2 and 1 edge respectively). This indicated that the high finding of non-embeddable matrices was not constrained to the initial gene tested. The parametric bootstrap was not carried out for the alignments for this second gene due to the computational and time demands.

### Phylogenetic Reconstruction

After establishing the existence of non-embeddable processes for sequence evolution, the focus changed to identifying any possible consequences of modelling a process (or multiple processes) as embeddable where this assumption may not be valid. The results of the phylogenetic reconstruction analysis reveal that the incorrect modelling of non-embeddable processes may have an impact on correct phylogenetic reconstruction. The results for each codon position and gene (D4) or species (D1) are shown in Table 10. Inconsistencies between the two models are again most present at codon position 3. For tetrads formed using sequences in dataset D3 almost twenty percent of the tetrads tested had differing probable trees at the third codon position. To test if these high findings were an artifact of the gene and possibly atypical, tetrads formed from alignments from a second gene, *nusA* (N utilization substance protein A), for the same 20 microbial sequences were tested for different probable trees using the same approach. The results for *nusA* returned much lower differences but in the same pattern with the third codon position providing the most inconsistencies. The tetrads formed using the mammalian dataset (D1) also revealed this pattern but at an extremely low level.

**Table 10:** Phylogenetic reconstruction results.

	Codon Position			
Species or Gene	1	2	3	Total Possible Tetrads or Alignments
*IF2^a^*	135	69	910	4845*^b^*
*numA^a^*	85	67	135	4845*^b^*
Mammalian	3	1	8	8005^c^

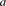
 – Microbial tetrads for 20 species (numA :N utilization substance protein A,IF2:translation initiation factor IF-2), 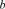 – Total Number of Tetrads , 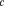 – Total Number of Alignments.

## Discussion

The purpose of identifying non-embeddability was to examine the validity of the common assumption of time-homogeneity by establishing cases where the assumption was violated. Violations were identified by establishing cases where non-embeddable matrices occurred and where a discrete model, making no assumptions about homogeneity, had a significantly better fit than a model assuming time-homogeneity. However, recently [40] demonstrated that under specific conditions there are instances where time-inhomogeneity can be accurately modelled by a time-homogeneous model.

For models that were multiplicatively closed it was demonstrated that it is possible for an inhomogeneous process to be precisely modelled as homogeneous [44]. They show that if a given Markov model 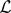 (such as the continuous model used here) forms a Lie algebra, and if the process is time-inhomogeneous e.g. described by two valid 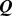 matrices that are in the model 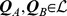 (with parameters allowed to be in the complex field), then there exists a matrix 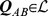 that can accurately describe the net process such that 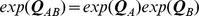. However it is possible that 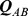 is not even stochastic if the rates have changed dramatically from 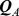 and 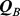 (as the case may be when modelling backward and forward time on the outgroup edge) or have parameters in the complex field (J. Sumner, personal communication, November 2012) meaning that that in these instances the process will be non-embeddable. It is possible that there are alignments that have been generated by a time-inhomogeneous process, but due to multiplicative closure of the continuous model used, that are embeddable. However should a similar study be carried out using the General Time Reversible (GTR) model, a model which is not multiplicatively closed, then all occurrences of time-homogeneity would cause non-embeddability. This may result in increased occurrences of non-embeddability and increase the chances of incorrect inference using such a model.

### Non-embeddability

Non-embeddability was identified for a number of alignments examined, indicating that for these alignments a time-homogeneous continuous model could not accurately model the underlying time-inhomogeneous process. Non-embeddable processes were generally found to occur on the outgroup edge or on edges with a large time depth and for protein-coding regions, at the third codon position. There was a clear difference in the number of non-embeddable matrices identified and the number of these subsequently classified as non-embeddable processes using the parametric bootstrap. Two possible causes are precision issues during the calculations and that the true process is actually embeddable or can be modelling accurately by an embeddable process.

Precision issues could cause the declaration of a non-embeddable 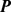 when in fact the true 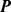 is embeddable. Precision issues were identified when examining 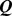 for negative off-diagonals; many matrices with extremely small negative entries were found. It was determined that these were artificial and most likely caused by precision during calculation. Consequently, a 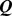 was only declared to have a negative off-diagonal element if the off-diagonal had a magnitude greater than 0.1. This was an arbitrary threshold based only on the presumption that this would exclude the majority of negative elements caused by any precision issues. However, precision issues are unlikely to be the major cause of the difference between the number of non-embeddable matrices and processes identified by the parametric bootstrap. The positive control revealed that 97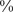 of the alignments simulated using a non-embeddable matrix were correctly identified as being generated by a non-embeddable process. This demonstrates that the approach has considerable power (for the generating conditions) to correctly identify processes generated by non-embeddable matrices using the parametric bootstrap.

**Figure 3:**
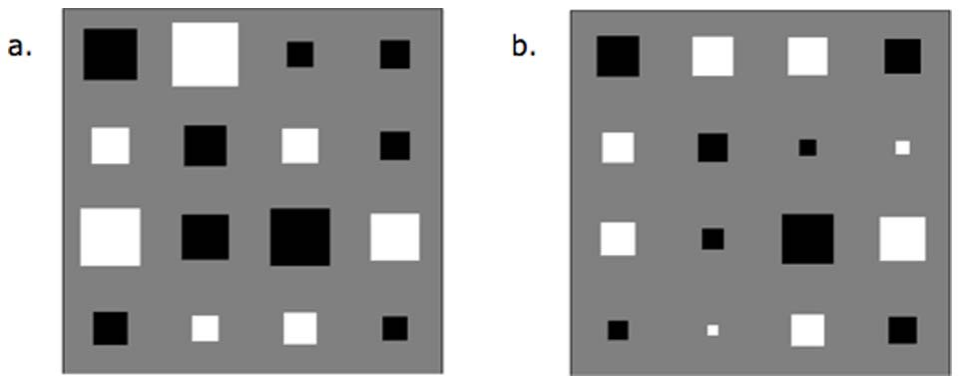
Average difference between matrices produced by the continuous (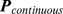) and discrete (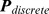) models (i.e. 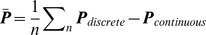) for alignments with non-embeddable (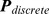) matrices which were found to be (a) Non-embeddable or (b) Embeddable using the parametric bootstrap. Where 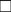 represents a larger transition probability in 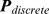 (e.g. 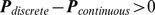) and ▪ indicates a larger transition probability in 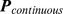 (e.g. 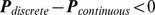).

Examination of the differences between the 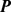 matrices produced assuming a discrete process (labelling this 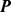 as 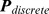) and assuming a continuous homogeneous process (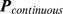) for an edge found to have a non-embeddable 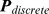 matrix revealed two distinct features. The first was that the closer together the two matrices were in 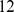 dimensional space the less likely the parametric bootstrap would find significant evidence of a difference in model fit indicating non-embeddability. The Frobenius norm (see Appendix) was used to measure the distance between 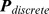 and 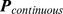 (e.g. 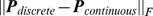). This distance was on average double for alignments where the parametric bootstrap found significant evidence of non-embeddability compared to those where no evidence was identified. For the opossum, mouse and human triad in the mammalian data set (D1) the average distance between 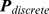 and 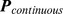, when 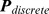 was non-embeddable, was 0.222 for alignments found to have significant evidence of non-embeddability in the parametric bootstrap compared to 0.117 for those without significant evidence. This suggests that if an embeddable 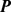 is close enough to the non-embeddable 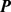 in 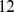 dimensional space then the embeddable 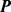 may be able to accurately model the process, explaining why the parametric bootstrap finds no significant difference in model fit. However, quantifying how close is close enough requires more investigation. This feature is also evident in Figure 3. Figure 3 displays, for the mammalian data set (D1), the average difference for 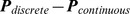, where 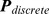 is non-embeddable, for processes found with significant evidence of being non-embeddable and those without evidence. The white represents a larger transition probability in the 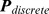 (i.e. 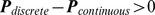) and black a larger transition probability in the 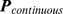 (i.e. 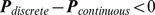). The alignments found to be embeddable using the parametric bootstrap can be seen to have smaller magnitude differences between 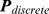 and 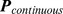.

The second distinct feature is that for an alignment with a non-embeddable 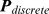 and significant evidence of a difference in model fit, there appears to have been an increased overall probability that a nucleotide will undergo a change to a different nucleotide. Examining the difference between the 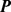 matrices (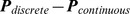) for non-embeddable processes shows that the 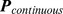 matrices are more diagonally dominant (see Figure 3). The 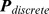 has larger off-diagonals (shown by larger white squares). As the rows of 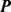 must add to one, in a non-embeddable 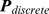 matrix there is, on average, an increased rate of nucleotide change.

For the observed non-embeddable matrices, we asked whether they are likely to have arisen from non-embeddable processes via parametric bootstrap. (Note that for embeddable matrices the question could not be evaluated even via parametric bootstrap because the likelihoods from the general continuous-time Markov equals, within precision, that from BH.) Across our datasets, the percentage of non-embeddable matrices that were indicative of non-embeddable processes at 5% significance varied from 0 to 12.1%. The most notable excesses are shown in Tables 6 and 8. For example, in Table 6, we observe that of the 99 non-embeddable matrices on the Human branch there was evidence of non-embeddable processes in 12 cases. This is in excess of the roughly 5% of cases in which we would expect to see significant results by chance alone. We conclude that non-embeddable processes exist amongst the cases where we observe non-embeddable matrices.

### Time depth is a determining factor for identifying Non-Embeddability

Time depth was expected to be a factor in identifying non-embeddability. Biologically, the greater the time depth on an edge the more likely a change in selection or mutation occurred requiring multiple 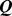 to model the process. Theoretically, when examining the matrix characteristics, for a continuous homogeneous process it is known that if 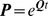 then 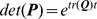 where 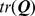 is the trace of the 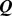 matrix (the sum of the diagonal entries or eigenvalues of 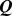). When 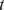 or 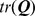 becomes large then 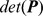 tends toward zero, which will result in a non-embeddable 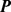. Hence, when 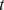 is large non-embeddable 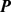 are more likely to be found. However, the number of matrices indicated as non-embeddable due to 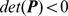 was significantly less than the number indicated by negative off-diagonals in 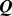 (Tables 4–9).

The impact of time depth on the occurrence of non-embeddability was primarily examined using the three Mammalian triads from the vertebrate data set (D1). Time depth is shown to be a determining factor for the identifying non-embeddability on both the ingroup and outgroup edges. This is clearly captured by examining the total number of non-embeddable processes for the three triads. An extremely low number of non-embeddable processes were identified for the Eutherian triad which has the shortest time depth between both the ingroup edges and the outgroup. The only evidence of non-embeddable processes were identified on the outgroup edge. However when the opossum taxa was used as the outgroup instead of the human taxa, this increase in time depth between the ingroup edges and the outgroup resulted in an increased number of non-embeddable processes established on the outgroup edge. The effect of deepening the time depth between ingroup taxa was examined using the opossum, human and mouse triad. This triad showed evidence of non-embeddable processes for a number of alignments on the ingroup edges.

The assertion that non-embeddability would be found on the outgroup edge is supported by datasets D1 and D3 (not examined in D4). Analysis of the primate intron data set (D3) showed only evidence of non-embeddability on the Macaque (outgroup) edge. Non-embeddability was indicated for 5 of the 62 alignments analysed. However in the mitochondrial dataset (D2), non-embeddability was indicated at the third codon position for mouse, an ingroup taxa. The results for the mitochondrial dataset must be interpreted with caution due to the small number of alignments and the possibility that the results may be confounded by the heterogeneous mutation rate found across the mitochondrial genome and genes e.g. [41]. This difference across sites rather than across time may confound the inferences and result in findings of non-embeddability on ingroup edges. However, if rate heterogeneity was causing false findings of non-embeddability across all datasets, then more evidence of non-embeddability would be expected to be found at the second codon position. The second position has been reported to exhibit the most rate heterogeneity [26]. In all datasets we considered there was no evidence of non-embeddability at the second position suggesting that rate heterogeneity is unlikely to be causing false findings of violations of the time-homogeneity assumption in the other datasets.

It is worth noting that the transition matrices from existing approaches to rate-heterogeneity are in fact non-embeddable. Several approaches to modelling rate heterogeneity have in common the specification of a transition matrix that is a weighted sum of other transition matrices. For instance, for the covarion model of Penny et al [42], one could construct a 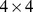 transition matrix by marginalising the 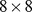 transition matrix. This 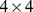 matrix is non-embeddable in general because it is a rather complicated combination of the two sets of underlying transition matrices. For discrete rates-across-sites models (e.g. discrete 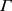, [43]), the transition matrix is a weighted average of 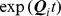, where the 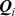
's are scaled versions of each other. For the continuous version (the 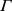 distribution, e.g. [44]), the weighted sum is replaced by an integral. In both cases, transition matrices are not embeddable. These three classes of models are robust to non-embeddability of the same kind as that implied by the models, i.e. when the true process and specified model are the same.

### Mutation And Not Selection Drives Non-Embeddability

Evidence from the protein coding datasets clearly show that violations of the time-homogeneity assumption are codon position sensitive. Non-embeddability principally affects the third codon position across the protein coding vertebrate and microbial datasets. If non-embeddability had been identified at codon position 2 this would likely indicate a change in the influence of selection, as all substitutions at codon position 2 causes amino-acid changes (non-synonymous substitutions). However evidence of non-embeddability was found predominately at the third codon position, the position that is the least constrained by selection for amino acids. Consequently the most likely cause is a change in the processes of mutagenesis. Additionally, evidence that non-embeddability also affects long (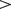
50000 bp) intron sequences (Table 8) further supports the notion that mutagenesis is the cause of the violation of the time homogeneous assumption, resulting in evidence of non-embeddable processes.

**Figure 4:**
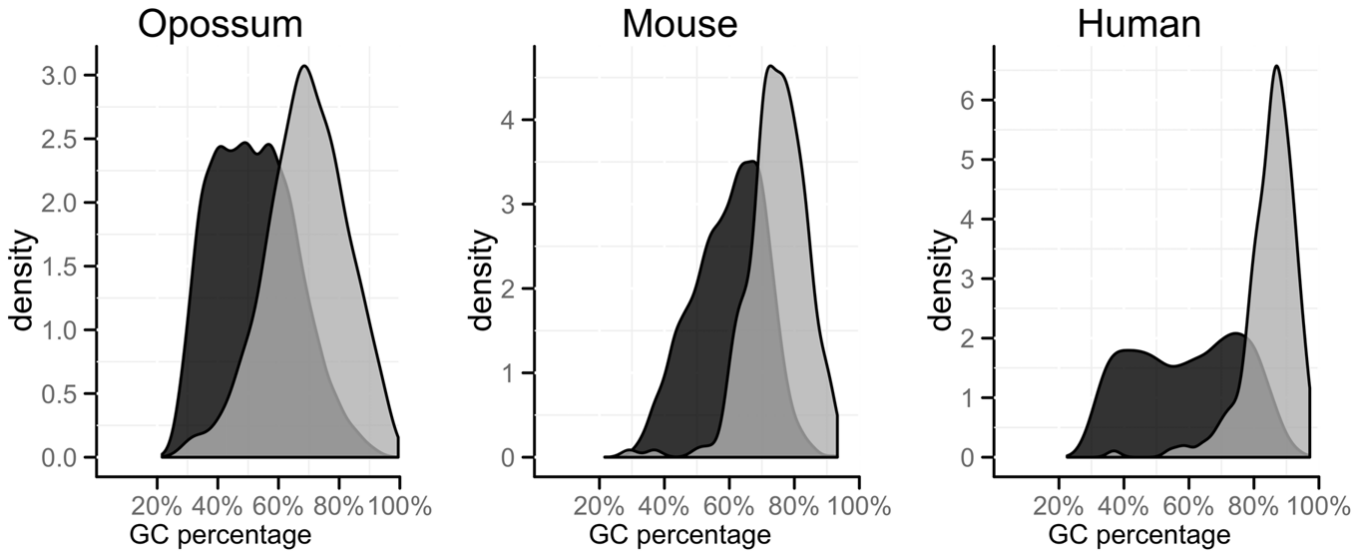
GC percentage for the Non-Embeddable (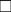) vs Embeddable (▪) Matrices for the Mouse, Human and Opossum Triad at the third codon position.

Examination of the features of the Mammalian alignments (D1) found that non-embeddable alignments revealed a strong GC bias at the third codon position. The alignments that the matrices indicated as non-embeddable had a significantly higher GC content at the third position than those indicated as embeddable (p-values 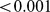 when comparing the GC content of the two groups). This feature is displayed for the mouse, human and opossum triad in Figure 4. The reason for this finding is not completely understood but it is likely that it is caused by biased-gene conversion [45]. In addition the GC content of alignments could be a candidate for driving mutagenesis. Observations that GC content is positively correlated with substitution rate [46], [47] suggest a link between regions of high GC and mutation rate.

### Non-embeddability affects Phylogenetic Reconstruction

Violations of the assumption of time homogeneity is shown to impact phylogenetic reconstruction. All codon positions appear to be affected, however, the third codon position appears the most problematic. These results coupled with the triad non-embeddability outcomes indicate that the third codon position is more susceptible to violations of the time-homogeneous assumption than either the first or second positions. To avoid possible violations of assumptions, the results indicate that using second codon position may provide the best option. However the degree of violation is dependent on the dataset; for example in the mammalian data set there were very few inconsistencies. This low number of inconsistencies may be a result of strong signals within the data indicating the phylogenetic structure for this tetrad. This means there is enough information for models (despite assumption violations) to correctly estimate the phylogenetic relationships. This is not always the case, as demonstrated in the microbial dataset.

There has been considerable debate about the use of the third position versus the first and second positions for phylogenetic reconstruction. This is because the first and second codon positions are considered to show less homoplasy (similarity due to convergent evolution). It was initially accepted that slowly evolving nucleotide sites were phylogenetically more informative than more rapidly evolving ones, especially for recognising more ancient groupings. For this reason third codon positions are often regarded as less reliable. However, [48] reported that “contrary to earlier expectations, increasing saturation and frequency of change actually improve the ability to recognize well-supported phylogenetic groups.” They concluded that eliminating third positions from phylogenetic analysis to be detrimental. However [49] reanalysed the same data and determined that while using the first and second position was a conservative approach, the phylogenetic groups supported by first and second positions, even if fewer in number, were compatible with those groups supported by third positions. The results presented here would suggest that the sampling of the second position while conservative would also avoid any possible violations of the time homogeneous assumption.

### Computing Issues

Optimisation can be problematic with such richly parameterised models and as such was specifically focused on during this study. A staged process of beginning with a simpler model e.g. globally time-homogeneous model, was found to increase the likelihood of convergence within a set number of maximum evaluations (normally 100 K). However as found within the microbial data set at the third codon position, convergence to a maximum within in the set number of evaluations did not ensure that the estimates found were stable. The mixed model was found at times to converge to different parameter estimates despite identical starting parameters provided by the global continuous model. Although this did change the parameter estimates provided to the discrete model, the discrete model was always able to converge to the same stable estimates. The reason for this may be that for these alignments especially at the third codon position, there was an extremely high amount of variation across the sequences (e.g. 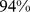 of sites were variable compared to 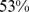 for the third position in the mammalian data set (D1)). The high number of variable sites appears to have resulted in the mixed model finding multiple local maxima, allowing the algorithm to exit before it reached the maximum number of evaluations. The discrete model was demonstrated to be more robust under these conditions.

Similarly a lack of information in alignments also affected optimisation. Short alignments and alignments with few variable nucleotides were found to be more likely to have unstable estimates as well as indicate non-embeddability and discrepancies between models (e.g. in mitochondrial data set (D2) when the sequence length was less than 150 bp). Alignments of reasonable length (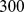
bp) but still with low number of variable sites between species were primarily found at the first and second positions and were also found to have unstable estimates.

## Conclusion

Violations of the local time-homogeneity assumption, evident through findings of non-embeddability, have been shown to exist when modelling sequence evolution with Markov models. Low levels of non-embeddability were detected when examining individual edges of triads across a diverse set of alignments. A deeper time depth between taxa increased the probability of a process being non-embeddable, while the outgroup edge was also shown to be the most likely to require multiple instantaneous rate matrices (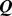) to describe the underlying process. Subsequent phylogenetic reconstruction analyses demonstrated that non-embeddability could impact on the correct establishment of phylogenies. However, the occurrence of inconsistencies was low.

While violations of the time homogeneity assumption appear to have minimal impact in some datasets, the existence of non-embeddability and possibility of any violations should be considered when modelling any evolutionary process.

## Supporting Information

Supporting Information S1Appendix A- The estimation of a valid 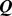 from a non-embeddable 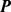 using the Frobenius norm.(PDF)
