# Supplementary material for: The Embedding Problem for Markov Models of Nucleotide Substitution
Source: PLoS One. 2013 Jul 30;8(7):e69187. doi: 10.1371/journal.pone.0069187 (PMC3728303; doi:10.1371/journal.pone.0069187)
Supplement: Supporting Information S1 — (PDF) [file pone.0069187.s001.pdf]

Appendix A

Finding a valid Q

Estimation of a valid Q from non-embeddable P matrices (P_{NE}) was done using a constrained optimisation procedure. The number of free parameters for any $\vec{Q}$ matrix was 12, equating to the number of off-diagonal elements of $\vec{Q}, q_{ij,i \neq j}$. This was because the diagonal elements were constrained to be $q_{ii} = -\sum_{j,j \neq i} q_{ij}$ to ensure that the row sums of $\vec{Q}$ were 0. The off-diagonals were constrained to be positive to ensure a valid $\vec{Q}$, $q_{ij,i \neq j} \geq 0$. The Frobenius norm was then used to find the nearest valid $\vec{Q}$. The Frobenius norm is the matrix norm of a $m \times n$ matrix defined as the square root of the sum of the absolute squares of its elements and is a measure of the effective size of a matrix given its elements e.g. for a matrix A the Frobenius Norm is:

$$||\vec{A}||_F = \sqrt{\sum_{i=1}^{m} \sum_{j=1}^{n} |a_{ij}|^2} \tag{1}$$

Thus to find the nearest valid $\vec{Q}$, $||\vec{P}_{NE} - exp(\vec{Q})||_F$ was minimised using optimisation procedures in PyCogent. The matrix exponential was computed by Pade.
